# Therapeutic host-directed strategies to improve outcome in tuberculosis

**DOI:** 10.1038/s41385-019-0226-5

**Published:** 2019-11-26

**Authors:** C. Young, G. Walzl, N. Du Plessis

**Affiliations:** South African Medical Research Council, Centre for Tuberculosis Research, Department of Science and Technology/DST-NRF Centre of Excellence for Biomedical Tuberculosis Research, Division of Molecular Biology and Human Genetics, Faculty of Medicine and Health Sciences, Stellenbosch University, Cape Town, South Africa

## Abstract

Bacille Calmette-Guérin (BCG) is the only licenced tuberculosis (TB) vaccine, but has limited efficacy against pulmonary TB disease development and modest protection against extrapulmonary TB. Preventative antibiotic treatment for *Mycobacterium tuberculosis* (*Mtb*) infections in high-prevalence settings is unfeasible due to unclear treatment durability, drug toxicity, logistical constraints related to directly observed treatment strategy (DOTS) and the lengthy treatment protocols. Together, these factors promote non-adherence, contributing to relapse and establishment of drug-resistant *Mtb* strains. Although antibiotic treatment of drug-susceptible *Mtb* is generally effective, drug-resistant TB has a treatment efficacy below 50% and can, in a proportion, develop into progressive, untreatable disease. Other immune compromising co-infections and/or co-morbidities require more complex prevention/treatment approaches, posing huge financial burdens to national health services. Novel TB treatment strategies, such as host-directed therapeutics, are required to complement pathogen-targeted approaches. Pre-clinical studies have highlighted promising candidates that enhance endogenous pathways and/or limit destructive host responses. This review discusses promising pre-clinical candidates and forerunning compounds at advanced stages of clinical investigation in TB host-directed therapeutic (HDT) efficacy trials. Such approaches are rationalized to improve outcome in TB and shorten treatment strategies.

## Abstract (web summary)

We present a comprehensive, up-to-date review of host-directed therapeutics (HDTs) to improve clinical outcome in tuberculosis (TB) disease, summarizing all compounds, primarily repurposed drugs, tested in human clinical trials. Where available, results from each phase of these investigations are discussed.

## Introduction

Tuberculosis (TB) remains the leading cause of death by infection worldwide.^1^ Despite introduction of directly observed treatment short-course (DOTS), the reduction in the global TB burden has been modest. The crisis is exacerbated by co-infections and co-morbidities, drug-resistant (DR) *Mycobacterium tuberculosis* (*Mtb*) strains and a rise in the reservoir of latent infection. Host immune status plays a determining role in TB disease outcome. It is also well-known that *Mtb* itself imposes several evasion strategies and prompts the host to elicit an immune response that favours its persistence. Adjunctive treatments aimed at “re-educating” the immune system are realistic alternative approaches to tailor host anti-TB responses. The use of host-directed therapeutics (HDTs) is intended to increase the success of TB treatment by immunomodulation and/or immune augmentation. Here, immunomodulation alludes to down-regulating non-productive inflammation and modifying the immune response. In contrast, immune augmentation is considered in the framework of synergizing with anti-TB treatment regimens of drug susceptible (DS)- and DR-TB to improve long-term outcome and promote cure.

HDTs are, therefore, considered crucial to achieving the 2035 World Health Organization (WHO) End TB goals.^2^ Repurposed compounds are more likely to be investigated in human clinical trials. In this regard, prior safety and regulatory approval increases the likelihood of fast-tracked implementation of drugs as appropriate immune response modifiers. Here we introduce HDT agents at advanced testing stages and highlight promising candidates for future HDT evaluations. These candidates may reveal favourable clinical outcomes and translate into useful adjunctive treatment strategies in our fight against TB.

## Host immune characteristics of TB

TB disease is perceived as a paradigm of host immune failure. In contrast, latency is considered a proxy of immunological control of *Mtb* infection. There is, however, no clear consensus of what constitutes clinically protective immunity. First-line innate immune defences play a central role in TB pathogenesis, albeit insufficient to clear infection. For this reason, T-helper (TH)-1 and CD8 T-cell adaptive responses are considered crucial for effective anti-TB immunity.^3,4^ Conversely, type-I interferon (IFN) and typical TH2 responses are associated with disease progression, contributing to disease susceptibility. Additionally, regulatory T cells (Tregs) may inhibit protective immunity.^5,6^ Theoretically, each of these pathways constitutes potential and ‘druggable’ targets. However, this notion is complicated by the complex course of progressive TB disease, including stages such as initial infection, protracted latency and overt disease^7,8^ Furthermore, other factors such as genetic diversity and co-morbidities (e.g. type-2 diabetes and HIV infection) also have a role to play.

A more recent concept is that TB represents a dynamic spectrum of mycobacteria at varying states of replication,^7^ highlighting the importance of immunotherapeutics treating the full TB spectrum. Realistically, a single immunotherapeutic agent is unlikely to be effective in the full TB spectrum. This has led to the concept of precision medicine approaches, since patient groups are likely to vary in their need for HDTs directed at immunomodulation and/or immune augmentation. For example, the treatment requirements from HDTs for individuals with advanced TB disease or even post-TB lung disorders are likely to differ considerably from those required for latently infected community members or healthy contacts of TB patients.

**Fig. 1:**
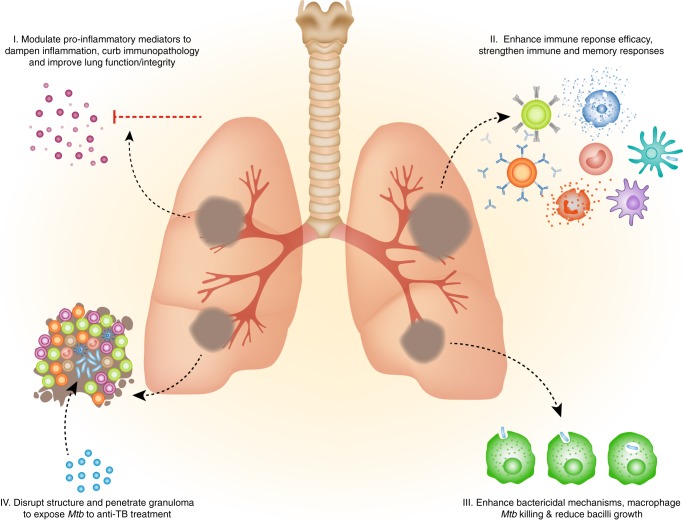
Main mechanisms by which repurposed, adjunctive compounds improve outcome in TB; **I** modulate inflammatory pathways and pro-inflammatory mediators to dampen inflammation and inflammation-induced tissue pathology and improve lung function/integrity, **II** enhance host immune response efficacy and strengthen immune and memory responses, **III** enhance host bactericidal mechanisms, macrophage-mediated *Mtb* killing and reducing bacilli growth, and **IV** disrupting and penetrating the granuloma to expose *Mtb* bacilli to anti-TB treatment.

The National Institutes of Health (NIH) clinicaltrials.gov resource database of privately and publicly funded human clinical trials lists investigations on adjunct therapies for various forms of TB. A literary search of human clinical trials, animal model studies and preliminary in vitro cohort studies was performed to identify current research highlighting repurposed drugs, HDTs and adjunctive candidates for TB treatment. Many of these have confirmed effective therapeutic manipulation of host immunity against *Mtb* and realignment of the response to support immune protection. Within the context of repurposed drugs, we summarize the four main mechanisms by which these adjunctive therapies are thought to improve outcome in TB (Fig. 1); namely, (1) mediating non-productive inflammation and inflammation-induced tissue pathology to improve lung function/integrity, (2) enhance host immune response efficacy and strengthen immune and memory responses, (3) enhance host bactericidal mechanisms, macrophage-mediated *Mtb* killing and reducing bacilli growth, and (4) disrupting and penetrating the granuloma to expose *Mtb* bacilli to anti-TB treatment.

## Promising TB HDT candidates tested in pre-clinical and human clinical trials

### Eicosanoid modulating drugs

Catabolism of arachidonic acid by cyclooxygenase (COX) enzymes produces prostaglandins, whereas lipoxygenase (LOX) metabolism yields leukotrienes.^9^ These eicosanoid products serve as signalling molecules, modulating inflammation and cell death. A delicate balance in eicosanoid levels is crucial for *Mtb* control and regulating the production of pro-inflammatory cytokines, such as tumour necrosis factor (TNF)-α (which plays a dual role in protection and exacerbated pathology in TB). Several modulators of the arachidonic acid pathway have been evaluated in humans as TB HDT.

#### Non-steroidal anti-inflammatory drugs (NSAID)

Emerging evidence shows that heightened levels of prostaglandins at late stages of *Mtb* infection (>45 days post-infection in mice) promote TB disease progression by down-regulating cell-mediated immunity.^10^ NSAID are commonly prescribed analgesic and anti-inflammatory medications worldwide and have shown promise as HDT in several pre-clinical studies.^11–13^ NSAID exert their effects by inhibiting COX activity, thereby interrupting formation of pro-inflammatory and immunosuppressive mediators such as prostaglandins and leukotrienes.^9,14^ Thus, the rationale for use of NSAID as HDT encompasses the inhibition of pro-inflammatory COX enzymes to attenuate excessive inflammation-induced tissue pathology and to improve host bactericidal mechanisms in individuals with active TB disease.

Resultantly, clinical trials have been initiated to assess the safety and efficacy of NSAID as adjunctive treatment of DS- and DR-TB (NCT02781909; NCT03092817; NCT02060006). For example, aspirin was investigated as an HDT candidate in a randomized trial during early TB treatment with dexamethasone in adult TB meningitis. Findings suggest that aspirin reduces new brain infarcts and related deaths through a mechanism involving inhibition of thromboxane-A2 and increased levels of protectins.^15^ Earlier trials also demonstrated reduced TB meningitis-associated strokes and mortality (Table 1).^16^ New generation NSAID selectively inhibiting the COX-2 enzyme are associated with less side-effects and gastrointestinal complications. Celecoxib and etoricoxib are currently undergoing evaluation in phase-I trials for safety and bactericidal activity in healthy volunteers and for efficacy as HDT in DS-TB (NCT02602509; NCT02503839; Table 1). Daily meloxicam, another selective COX-2 inhibitor, is currently under investigation in a randomized control trial, for its ability to prevent development and severity of paradoxical TB immune reconstitution inflammatory syndrome (TB-IRIS) (NCT02060006). The WHO recommends routine inclusion of NSAID as an adjunctive therapy to the standard TB treatment regimen to reduce antibiotic-related joint pain. However, the use of NSAID as preventative treatment remains unclear. Compared with nonusers, use of traditional NSAID was associated with an increased risk of TB in an unadjusted analysis of a population-based study.^17^ Results from carefully controlled trials should provide more conclusive findings on the effect of these inexpensive and widely available compounds on TB treatment outcomes.

**Table 1:** Clinical trials investigating adjunctive therapies in TB disease.

Adjunctive HDT	Rationale for use	Hypothesized clinical benefit	Clinical trial study title	Phase	Location	Primary outcomes evaluated	Reference
Nonsteroidal anti-inflammatory drugs (NSAIDs)							
Ibuprofen	NSAIDs exert their anti-inflammatory properties by inhibiting COX-1 and COX-2 cyclooxygenases, which in turn, modulate proinflammatory, immunosuppressive mediators such as prostaglandins and leukotrienes. By inhibiting the effects of cyclooxygenase enzymes, chronic host inflammatory responses leading to pathological lung lesions are halted, while bactericidal mechanisms and vaccine response of the immune response are increased.	Mediate non-productive inflammation and inhibit inflammation-induced tissue pathology	Potential Efficacy and Safety of Using Adjunctive Ibuprofen for XDR-TB Tuberculosis	2	Georgia, USA	Microbiological, radiological and clinical efficacy-related events.	^131–133^
Aspirin			A Pilot Study of Adjunctive Aspirin for the Treatment of HIV Negative Adults with Tuberculosis Meningitis	2	Ho Chi Minh City, Vietnam	Episodes of cerebral or upper-gastrointestinal bleeding; episodes of MRI-proven brain infarction or death.	^134,135^
Etoricoxib			Therapeutic Vaccination and Immune Modulation – New Treatments Strategies for the MDR Tuberculosis Pandemic	1	Oslo, Norway	Safety and immunogenicity (total CD4 T-cell cytokine responses; IFN-γ, IL-2, TNF-α).	^136^
Meloxicam			TB-IRIS NSAID Cox-2 Inhibitor Prevention Trial	3	Cape Town, SA	Incident of TB IRIS.	
Celecoxib			Evaluating Celecoxib Activity in Mycobacterium Tuberculosis: A Whole Blood Bactericidal Activity Study in Healthy Volunteers	1	Singapore	Log change CFU/day calculated from MGIT time to positivity	^137,138^
Vitamins and dietary supplements							
Vitamin A	Vitamin A is essential for regular immune functions, where a deficiency is correlated with increased mortality in HIV/TB co-infected individuals. Supplementation of vitamin A is thought to strengthen the immune system and reduce morbidity.	Enhance host immune response efficiency and strengthen immune system	Vitamin A Therapy for Tuberculosis	3	Zomba, Malawi	Mortality and morbidity	^139^
Cholecalciferol / Vitamin D	Vitamin D acts via its receptor (VDR) found in activated immune cells and regulates gene expression of cytokines and mediators of the immune response. During the antimicrobial immune response, VDR is upregulated following ligation of TLRs, which induces cathelicidin LL-37 and β defensins, which aid in bacterial killing as antimicrobial peptides. Thus, supplementation of vitamin D as an adjunct therapy is thought to enhance the immune response and result in favourable disease outcome.		Trial of Adjunctive Vitamin D in Tuberculosis Treatment	3	London, UK	Time to sputum culture conversion.	^140–144^
			Effect of Vitamin D as Adjunctive Therapy in Patients with Pulmonary Evolution Tuberculosis	4	Mexico City, Mexico	Determination of cytokines (IFN-γ, IL-17, TNF-α).	
			TB Host Directed Therapy (TBHDT)	2	Gauteng, SA	Adverse events and sputum culture conversion.	
			Pulmonary Tuberculosis and Vitamin D	3	New Delhi, India	Sputum culture conversion.	
l-Arginine and vitamin D	Targets the two major macrophage killing pathways namely, arginine-nitric oxide and vitamin D-1,25 dihydroxyvitamin D pathways, to improve the magnitude and promptness of the immune response.		l-arginine and Vitamin D Adjunctive Therapy in Pulmonary Tuberculosis (TB)	3	Timika, Indonesia	Sputum culture conversion, improvement in composite clinical endpoint (weight, cough clearance, FEV1).	^145,146^
Phenylbutyrate and Vitamin D	Sodium 4-phenylbutyrate (PBA) acts in synergy with vitamin D metabolites to upregulate the expression of cathelicidin antimicrobial peptide, which augments *Mtb* growth, while induces antimicrobial and anti-inflammatory responses in favour of the host.		Clinical Trial of Phenylbutyrate and Vitamin D in Tuberculosis (TB)	2	Dhaka, Bangladesh	Sputum culture conversion, clinical endpoints (cough clearance, chest X-ray clearance, fever, weight).	^147,148^
			Immune Reconstitution in Tuberculosis Disease	2	Lideta, Ethiopia	Composite clinical TB score.	
Zinc	Malnutrition and zinc deficiency diminishes antibody- and cell-mediated immune responses, which is detrimental to the host in TB disease. Supplementation of zinc micronutrients are thought to increase the efficacy of the immune response against TB infection by protecting cells against the pathological effects of free radicals and hypoxia-induced lung damage.		Efficacy of Oral Zinc Administration as an Adjunct Therapy in New Pulmonary Tuberculosis (Category I) Patients	3	New Delhi, India	Sputum culture conversion, efficacy, relapse rate, adverse events.	^149–151^
Immunoxel/Dzherelo	Immunoxel is an oral immunomodulatory botanical compound that strengthens the immune system and aids in the ability of the host to clear infectious diseases by restoring humoral and cellular immunity and increasing interferon production.		Adjunct Immunotherapy with Immunoxel in Patients with TB and TB/HIV	3	Kharkiv, Ukraine	Efficacy, safety, effects on lymphocytes and CD4 T cells, CD4/CD8 ratios	^152–155^
Corticosteroids							
Prednisolone	Corticosteroids exert their anti-inflammatory effects by binding to intracellular receptors and modulate gene transcription in target tissues, thereby interfering with the function of inflammatory mediators, suppressing humoral immune responses and inhibiting leukocyte infiltration at the site of disease. The anti-inflammatory actions involve phospholipase A2 inhibitory proteins, which control prostaglandin and leukotriene synthesis. This anti-inflammatory responses in TB is thought to reduce chronic inflammation-induced tissue pathology and modulate the immune response in favour of the host.	Mediate non-productive inflammation and inhibit inflammation-induced tissue pathology	A Trial of Adjunctive Prednisolone and Mycobacterium w Immunotherapy in Tuberculosis Pericarditis	3	Cape Town, SA	Composite end-point of death, constriction, cardiac tamponade requiring pericardial drainage.	^156–159^
			Corticosteroids in the Treatment of Tuberculous Pleurisy	N/A	Guangxi, China	Death, pleural thickening, pulmonary function, adverse events.	
			Tuberculosis in HIV Infected Patients in Uganda	2	Kampala, Uganda	Not available.	
Dexamethasone			Adjunctive Corticosteroids for Tuberculosis Meningitis in HIV-Infected Adults (The ACT HIV Trial)	3	Jakarta, Indonesia	Survival, neurological disability.	^160–166^
			Leukotriene A4 Hydrolase Stratified Trial of Adjunctive Corticosteroids for HIV-uninfected Adults With Tuberculosis Meningitis	3	Ho Chi Minh City, Vietnam	Mortality, neurological events, adverse events.	
			The Relationship Between Gene Polymorphisms of LTA4H and Dexamethasone Treatment for Tuberculosis Meningitis	4	Hebei, China	MRI evidence of infarction, hydrocephalus, meningeal enhancement; changes in cerebrospinal fluid and clinical symptoms.	
Other drugs as adjunctive TB treatment							
Pravastatin	Statins act as inhibitors of HMG-CoA reductase enzymes, which express lipid-lowering, immunomodulatory and anti-inflammatory activities, and have been shown to reduce bacterial growth and express anti-TB activity in macrophages. This is achieved by reducing infection-induced lipid accumulation in macrophages and enhancing phagosome maturation and autophagy.	Mediate nonproductive inflammation and tissue damage, enhance host bactericidal mechanisms by promoting macrophage-mediated killing of *Mtb* and reducing bacterial growth	Statin Adjunctive Therapy for TB (StAT-TB)	2	Not available	Safety of escalating doses of pravastatin co-administered with rifampin.	^81,82,167^
Auranofin	Auranofin is an organogold compound that induces transcription of heme oxygenase 1 (HO-1) mRNA. HO-1 is an inducible heme-degrading enzyme with anti-inflammatory properties which acts to suppress the immune response and decrease free radical production, thus enhancing oxygen-mediated killing and exerting bactericidal activity in TB disease.		TB Host Directed Therapy (TBHDT)	2	Gauteng, SA	Adverse events and sputum culture conversion.	^168,169^
CC-11050	CC-11050 inhibits phosphodieasterase-4 (PDE-4) and acts as an anti-inflammatory compound that modulates chronic inflammation and cytokine storms associated with infectious diseases, while improving antibiotic response and reducing bacillary load in the host.		TB Host Directed Therapy (TBHDT)	2	Gauteng, SA	Adverse events and sputum culture conversion.	^28,170–172^
*N*-acetylcysteine	Cleavage of the acetyl group of *N*-acetylcysteine makes cysteine available for incorporation of the glutathione synthesis pathway. The antioxidant effects modulate chronic inflammation, exhibit anti-mycobacterial activity and improve disease outcome.		RIPE vs. RIPE Plus *N*-acetylcysteine in Patients With HIV/TB Co-infection	2	Manaus, Brazil	Biological intolerability, adverse events, culture conversion, hepatotoxicity, dosage.	^173^
Imatinib	Imatinib, a tyrosine kinase inhibitor, promotes myelopoiesis, phagosome maturation, acidification and autophagy to reduce bacillary burden of *Mtb*.		A Phase II Clinical Trial of the Safety, Pharmacokinetics and Hematologic Effects of Imatinib on Myelopoiesis in Adults When Given With and Without Isoniazid and Rifabutin	2	Not available	Changes in the number of myelomonocytic cells in the blood and adverse events	^36,38–41^
Doxycycline	Doxycycline is a tetracycline antibiotic that dampens host inflammatory responses by inhibiting MMPs, which typically degrade collagen and structural proteins to induce tissue damage and cavitation.		Doxycycline in Human Pulmonary Tuberculosis (Doxy-TB)	2	Singapore	Change of serum marker Procollagen III N-terminal peptide (PIIINP) and adverse events	^96,97^

*Mtb Mycobacterium tuberculosis*, *TB* tuberculosis *NSAIDs* nonsteroidal anti-inflammatory drugs, *COX* cyclooxygenase, *HIV* human immunodeficiency virus, *VDR* vitamin D receptor, *TLR* toll-like receptor, *PBA* sodium 4-phenylbutyrate, *CD* cluster of differentiation, *IL* interleukin, *DNA* deoxyribonucleic acid, *RNA* ribonucleic acid, *HMG-CoA* 3-hydroxy-3-methylglutaryl-CoA, *HO-1* heme oxygenase-1, *mRNA* messenger ribonucleic acid, *PDE-4* phosphodiesterase-4, *FEV* forced expiratory volume, *MMPs* matrix metallopeptidases

This table gives a summary of all completed and currently active clinical trials investigating adjunctive HDT options in TB, with references to supporting preclinical investigations

#### Lipoxygenase inhibitors

Eicosanoids were previously suggested as targets for therapeutic exclusion in TB. However, data demonstrate the protective role of prostaglandin E (PGE)-2 during early infection, either by direct supplementation or via inhibition of 5-Lipoxygenase (5-LOX). Inhibiting 5-LOX has been linked to restricted lung pathology, lower type-I IFN production, reduced *Mtb* replication and greater survival rates in a TB-susceptible murine model^18^; thus rationalizing the prospective use as adjunctive therapy to improve TB outcome. Accordingly, individuals with latent TB, who fail to develop active TB disease, display balanced levels of PGE-2 and lipoxins.^18^ Lipoxins also negatively regulate protective TH1 responses. This was demonstrated by increased IFN-γ, interleukin (IL)-12 and nitric oxide synthase (NOS)-2 mRNA levels and reduced mycobacterial burden in 5-LOX-deficient mice.^19^ At present, no trials evaluating 5-LOX inhibitors as HDT complementing standard TB treatment are registered on the clinicaltrials.gov resource database. The 5-LOX inhibitor, zileuton, is however, approved for treating asthma which could be repurposed as TB HDT and tested to elucidate whether modulation of this pathway improves TB treatment outcomes.

### Inflammatory modulators

#### Corticosteroids

Corticosteroids have been employed as adjunctive therapy for a range of inflammatory conditions and disease states, including bacterial and viral meningitis, pneumonia and sepsis.^20,21^ In TB, hyper-activation of the inflammatory response often results in tissue pathology and oedema, leading to tissue dysfunction and chronic inflammation. The rationale for using anti-inflammatory corticosteroids as adjunctive treatment of active TB disease mechanistically involves modulation of inflammatory and apoptotic gene transcription pathways.^22^ This occurs by binding to intracellular receptors and modulating gene transcription in target tissues, thus modulating inflammatory mediator function, suppressing the humoral immune response and inhibiting leucocyte infiltration to the site of disease.^23,24^ These effects are thought to reduce chronic, non-productive inflammation and favour the host antimicrobial response.

Corticosteroids as immunoadjuvants to standard TB treatment have proven useful in several studies, including an HIV/TB co-infection framework. Supporting evidence demonstrates improved lung radiological lesions, earlier symptomatic improvement and reduced morbidity in severe disease.^25–28^ In particular, trials testing the efficacy of adjunctive dexamethasone treatment on the risk of death or disability in TB meningitis demonstrated improved patient survival rate (Table 1).^29^ Other phase-III and IV multicentre trials, investigating survival and disability outcomes following dexamethasone adjunctive treatment of TB meningitis, are underway (NCT03100786; NCT03092817; NCT02588196; Table 1). Similarly, prednisolone for treating TB pericarditis in HIV infection was investigated in a phase-III trial (NCT00810849; Table 1). Results indicate no significant effect on the combined outcome of death, cardiac tamponade or constrictive pericarditis, although prednisolone did reduce incidences of pericardial constriction and hospitalization.^30^

Importantly, since data suggest that the effects and benefits of corticosteroid adjunctive therapy are organ specific, its use in extrapulmonary TB requires careful consideration on a case-specific basis. Meta-analyses refute long-term treatment efficacy of corticosteroids. In fact, studies involving high-dose corticosteroid treatment observed an increased risk of side effects.^31^ Low-dose trials, however, appear to circumvent such consequences, while maintaining favourable clinical outcomes in pulmonary TB (PTB) disease.^25,31,32^ Taken together, it is evident that more investigation is needed to establish conclusive outcomes for the risks and benefits of corticosteroids as adjunctive therapy for advanced TB disease.

#### Phosphodiesterase inhibitors

Phosphodiesterase inhibitors (PDE-i) are small-molecule inhibitors that reduce inflammation by increasing intracellular cyclic adenosine monophosphate (cAMP) and cGMP.^33^ Altogether, the anti-inflammatory effects of PDE-i serve to modulate chronic inflammation and cytokine storms associated with infectious disease, while improving antibacterial responses and reducing bacillary load. This suggests relevance, not only during active TB disease, but also in clearing non-productive inflammation for conditions such as TB-IRIS and extrapulmonary TB.

Several selective PDE-i have shown promise as HDT candidates in TB animal models. Inhibitors of PDE-3 and PDE-5, cilostazol and sildenafil respectively, accelerated bacterial clearance and lung sterilization in murine TB.^34^ The PDE-4-i, roflumilast has also shown promise as an effective HDT in a TB mouse model when used with isoniazid. Supporting evidence illustrated reduced TNF-α production, thwarted neutrophil recruitment and reduced lung bacillary burden.^35^ Similar findings were reported for another selective PDE-4-i, CC-11050, in a TB rabbit model.^36^ Analogues of thalidomide, such as CC-3052, have also shown to possess similar PDE-4-i properties and demonstrated potential as TB HDT by reducing lung pathology and inflammation.^37^ These promising pre-clinical screenings of PDE-i have led to safety and efficacy testing of adjunctive CC-11050 with the standard 6-month multi-drug therapy. This phase-II open-label human clinical trial is currently recruiting South African TB patients (NCT02968927; Table 1). Pending outcome of these results, several other members of the PDE-i family represent attractive HDT candidates. These include PDE-5-i, shown to reverse the host immunosuppressive effects of regulatory immune cells such as myeloid-derived suppressor cells (MDSC) in cancer.^38^

#### *N*-Acetylcysteine (NAC)

NAC is an l-cysteine prodrug, which replenishes levels of the antioxidant glutathione by making cysteine available for incorporation into the glutathione synthesis pathway. NAC, often prescribed to patients with chronic pulmonary disease, has mucolytic and antioxidant activities, with the capacity to modulate inflammation.^39^ In vitro data rationalizing improved outcome in TB indicate a dose-dependent NAC-mediated reduction in *Mtb* growth and metabolic activity. This occurs by suppressing the host oxidative response, along with direct anti-mycobacterial affects.^40,41^ Therefore, beneficial effects of NAC as HDT is not limited to use in symptomatic TB disease and post-TB lung disease, but also to clear *Mtb* in healthy latently infected individuals.

NAC has subsequently been tested in a prospective randomized control trial, demonstrating significantly faster sputum conversion with improved lung pathology in TB patients receiving daily NAC treatment during the intensive phase of DOTS.^42^ Additionally, NAC has a hepatoprotective effect on liver injury during TB treatment (Table 1),^43,44^ and a murine macrophage model has illustrated the ability of NAC to potentiate the efficacy of TB chemotherapy, specifically in combination with isoniazid.^46^ Together, these data provide promising outlooks for NAC as an adjunctive therapy to synergize with current therapies and improve outcome in TB. Currently, a phase 2 randomized trial is investigating the tolerability and treatment outcome of daily adjunctive NAC for 2 months in conjunction with the standard 6-month TB treatment regimen in Brazil (NCT03281226). Outcomes from ongoing studies should provide greater insight and justification for further trials investigating concomitant NAC for treatment of multi-drug-resistant (MDR)-TB patients (Table 1).

### Tyrosine kinase inhibitors

Another avenue for treating MDR-TB and HIV/TB co-infection includes the use of tyrosine kinase inhibitors as HDT. Imatinib is a tyrosine kinase inhibitor typically employed for treatment of cancers, more specifically, chronic myelogenous leukaemia. In the context of *Mtb* infection, beneficial outcomes of imatinib are associated with reducing bacillary burden by promoting myelopoiesis, phagosome maturation and acidification, and autophagy.^45,47–49^ Findings have illustrated that imatinib as an adjunctive therapy with first-line anti-TB drugs has synergistic therapeutic effects.^48,49^ A study by Steiger and colleagues in 2016 showed that imatinib induced lysosome acidification and antimicrobial activity against *M. bovis* in human macrophages treated with glucocorticoids. Notably, these effects were exhibited without reversing the anti-inflammatory effects of glucocorticoids.^50^ A clinical trial (NCT03891901; Table 1) is scheduled to roll out soon, which aims to evaluate safety, pharmacokinetics and effects of imatinib on myelopoiesis in adults, as a potential adjunctive therapy with an antimicrobial regimen for DS-TB.

Other tyrosine kinase inhibitors have shown potential in in vitro and murine studies. One candidate specifically, geftinib, an FDA-approved inhibitor of epidermal growth factor receptor (EGFR) tyrosine kinase, has shown promise in both acute and chronic *Mtb* infection, by augmenting TH1 immunity and reducing bacterial load.^51^ Tyrosine kinase inhibitors that have been studied extensively in tumour models, such as nilotinib, are now showing promising pharmacological expansion of protective innate immunity to mycobacterial infections.^52^ These compounds are attractive candidates for testing as a prophylactic anti-TB regimen in high-risk communities.

### Antihyperglycaemic drugs

Poor glycaemic control is a risk factor for TB disease onset, mortality, treatment failure and relapse.^53,54^ In this regard, metformin (class: biguanide) treatment improves glucose control in diabetic patients and restores dysfunctional immunity associated with hyperglycaemia.^55–57^ In the context of TB, the immunomodulatory effects of metformin have been shown to promote macrophage autophagy by activating the expression of AMP-activated protein kinase (AMPK) and reactive oxygen species (ROS) production. Altogether these effects inhibit *Mtb* growth, reduce inflammation and prevent lung damage.^58^ These findings have promoted metformin as a candidate for therapeutic prevention and adjunctive treatment approaches in TB.^55^

In vitro and in vivo murine studies have further shown that metformin treatment reduces inflammation in TB by promoting expansion of anti-inflammatory cell types, particularly alternatively activated macrophages.^59–62^ A number of pre-clinical studies further demonstrated that metformin synergizes with the antimicrobial properties of rifampicin and reduces intracellular *Mtb* growth. This occurs in an AMPK-dependant manner, through inhibition of pro-inflammatory cell proliferation, thereby reducing disease severity.^63,64^ Other in vitro and in vivo findings show that metformin promotes ROS production, required for fusion of the phagosome-lysosome complex to aid in phagocytosis- and autophagy-induced killing of *Mtb*.^64,65^ Metformin also has a direct effect on the bacterial respiratory chain complex, which plays an important role in bacterial persistence and tolerance.^66^ Retrospective evaluation of clinical trial data demonstrated that metformin treatment of type-II diabetics with TB is associated with fewer lung cavities, lower proportion of individuals with advanced disease and improved sputum culture conversion rate 2 months post-treatment initiation.^58,64,67^ Retrospective analyses also showed that type-II diabetics receiving metformin treatment have a lower TB risk profile compared to those using sulfonylureas.^68^ Interestingly, diabetics on metformin treatment have a lower chance of having latent *Mtb* infection (LTBI), as measured by a positive T-Spot TB test.^64^

Most clinical testing has been limited to evaluating the effects of metformin on diabetic TB patients. This begs the question of whether these same properties translate to non-diabetic TB patients in a clinical setting. Pre-clinical evidence of a recent in vitro study showed metformin-mediated modulation of cellular metabolism, immune function and gene transcription involved in innate immune responses to *Mtb* in heathy subjects.^69^ Moreover, metformin has illustrated beneficial effects for non-diabetic indications such as obesity, polycystic ovary syndrome and Alzheimer’s disease.^70^ The first prospective trial testing the addition of metformin to DOTS, the ‘Metformin for TB/HIV Host-directed Therapy’ (METHOD) trial, is currently in planning and will evaluate the proportion of smear negative TB patients at month two post-treatment. This trial will also test the efficacy of metformin on lung function, severity of lung involvement and HIV viral load (R34-AI124826-01).

While the TB community is anxiously awaiting results from clinical trials testing these and other drugs with anti-inflammatory or antihyperglycemic properties, it remains important to carefully consider and assess ideal dosages to prevent excessive anti-inflammatory responses, which too may favour *Mtb* proliferation.

### Vitamins and biologics

Vitamins are furthest along the pipeline of TB HDT testing in human clinical trials (Table 1). This is likely attributed to the relative ease of accessibility and low risks associated with vitamin supplementation. Vitamins are essential for regular immune function and deficiencies have been implicated in a range of disease states. As such, vitamin supplementation may be applicable as an adjunctive therapy to the standard of care to improve outcome to TB disease. On the other hand, perhaps vitamin supplementation may pose as a preventative strategy to strengthen the immune system and prevent progressive onset of disease.

Vitamin D (vitD), acting via its vitD receptor (VDR), regulates gene expression of cytokines and immune mediators in activated cells. In the antimicrobial immune response, VDR is upregulated following ligation of TLRs, which induces antimicrobial peptides such as cathelicidins and defensins. Thus, vitD as an adjunctive therapy may enhance the immune response and favourable disease outcome in TB. At least 22 current and completed trials of vitD as TB HDT are listed on the clinicaltrials.gov database. Inconsistencies in trial outcomes have, however, impeded interpretation of HDT efficacy. While some studies on vitD supplementation during TB treatment demonstrate clinical and radiological involvement in patients with vitD deficiency (Table 1), others fail to show any advantage on TB outcomes (Table 1).^71,72^ At a pre-clinical level, the protective effects of vitD has been linked to enhanced innate immune production of ROS, IL-1β, IFN-γ and cathelicidin,^73,74^ while positive trials have shown a reduction in inflammatory mediators including matrix metalloproteinases (MMPs) (Table 1). It is believed that contrasting trial outcomes reflect variations in vitD administration and dosage, differing levels of endogenous baseline vitD, genetic differences in the vitD receptor, underpowered cohorts and variations in sunlight exposure at trial locations.

Vitamin A (vitA) deficiency has also been associated with incident TB and correlated with increased mortality in HIV/TB co-infected individuals. VitA supplementation is thought to strengthen the immune system and reduce mortality; however, information on vitA supplementation in conjunction with TB treatment has been inconsistent. The handful of completed case-control studies investigating vitA supplementation during TB treatment mainly report findings as part of a dietary multivitamin supplement.^75^ Therefore, evidence of the direct benefit of vitA has been weak, at best demonstrating modest improvements in the weight of TB patients.^76^ VitA supplementation with zinc yielded similar results^75^ (Table 1). A more recent case control study, nested within a longitudinal TB household contact study, showed that baseline vitA deficiency was associated with a tenfold increased risk of developing TB.^77^

Although the overall findings in the area of vitamins as TB HDT is promising, significant challenges exist that impede objective interpretation of data. This mainly stems from heterogeneous study design with discrepancies in nutritional/dietary intake and route of administration, amongst others. We propose that meticulous study design may overcome these challenges and may provide more conclusive data for the use of vitamin supplementation to improve TB outcome.

Sodium phenylbutyrate (PBA), a biological aromatic fatty acid, has been approved for treating various diseases, including urea cycle disorders, cancer, muscular dystrophy and Parkinson’s disease. Its functions include inhibition of histone deacetylase and endoplasmic reticulum stress.^78,79^ Pre-clinical studies and clinical trial data have shown that PBA synergises with vitD to upregulate expression of the anti-mycobacterial peptide cathelicidin, restrict *Mtb* uptake, reduce *Mtb* intracellular growth in macrophages, upregulate chemokine secretion and induce autophagy.^80,81^ These benefits of PBA has been verified in a randomized controlled trial in Bangladesh, demonstrating its potential as TB HDT (Table 1).

Other immunomodulatory biologics include immune checkpoint inhibitors (ICIs). The ICIs currently attracting the most attention in TB include nivolumab and ipilimumab. Nivolumab is a monoclonal antibody targeted against programmed death (PD) 1 protein, while ipilimumab targets cytotoxic T-lymphocyte-associated antigen 4 (CTLA4). Typically, signalling via immune checkpoints inhibits T- and B-cell function. However, in the context of TB, immune regulatory checkpoints are dysregulated and associated with T-cell exhaustion.^82–86^ Despite promising outcomes in animal and in vitro models, clinical use of ICIs may favour progression to active TB disease, potentially attributed to excessive inflammation and focal necrosis.^87^ Such therapies thus require careful consideration regarding method, dose and timing of administration to minimise potential negative effects.

### Cytokine modulation

Therapeutic modulation of immunity via cytokines is another method to support host defences. Cytokines play crucial roles in immune cell function and can theoretically serve as promising candidates for inclusion in adjunctive immunotherapies. This, however, is strictly dependent on role of a given cytokine in host immunity. Reducing excessive cytokine responses appears as a promising HDT strategy for individuals with active DR- and DS-TB.^88,89^ In contrast, boosting TH cytokine responses could serve as a feasible strategy for those with acute/recent *Mtb* infection.^90,91^

Cytokines may polarize the immune response in favour of host protection by strengthening immune and memory responses or by disrupting and penetrating the granuloma to expose *Mtb* bacilli to anti-TB treatment (Table 2; Fig. 1). The hallmark TH1 cytokines, namely IFN-γ, IL-2, IL-12 and GM-CSF, have been highlighted as recombinant therapeutics in adjunctive HDT trials. Additionally, the activity of TH2/immunosuppressive cytokines may be modulated as an alternative strategy^92–96^ (Table 2). A currently active, phase-II interventional trial of pascolizumab, an anti-IL4 monoclonal antibody, is being investigated in TB patients receiving standard treatment (NCT01638520; Table 1). As the TB community eagerly awaits this outcome, another randomized, placebo-controlled trial disappointingly demonstrated that adjunctive recombinant IL-2 immunotherapy in TB patients did not afford a statistically significant improvement in bacterial clearance as measured by culture conversion at months 1 and 2^97^ (Table 2).

**Table 2:** The role of cytokines in TB disease and evidence to support their therapeutic intervention and outcome as TB HDT strategies.

Cytokine	Role in TB disease	Therapeutic intervention and outcome in TB disease	Reference
INF-γ	Activates macrophages and DCs, promotes cell proliferation, apoptosis, cell adhesion and bacterial killing (through phagocytosis and reactive nitrogen and oxygen intermediates).	Aerosol administration of INF-γ improved bacillary clearance and improved clinical condition. Aerosol administration of INF-γ in conjunction with anti-TB drugs cured MDR-TB. Nebulized INF-γ1b and subcutaneous injections of INF-γ1b alleviated disease symptoms, although culture showed that nebulized administration increased the likelihood of negative smears at 4 weeks. Supplementation of recombinant INF-γ improves response to anti-TB drugs in cavitary TB patients.	^75,174–177^
TNF-α	Controls *Mtb* infection and replication by formation and maintenance of the granuloma; by regulating macrophage activation, phagocytosis, and nitrogen and oxygen intermediates.	TNFα inhibition causes granuloma disruption and bacillus reactivation to increase *Mtb* susceptibility to standard TB drugs; results in rapid clearance of *Mtb* from the lung and altered inflammatory responses to benefit the host in TB/HIV-1 co-infected individuals.	^178,179^
GM-CSF	Induces granulocyte and macrophage proliferation and differentiation, stimulates macrophage phagocytosis, increases cytotoxicity and reactive nitrogen and oxygen intermediates.	Administration of GM-CSF resulted in negative sputum culture conversion after 8 weeks.	
TGF-β*	Immunosuppressive cytokine, inhibits the Th1 response during chronic infection.	Suppression of TGF-β enhances resolution of local *Mtb*infection and associated inflammatory responses, while decreasing bacillary load in mouse models.	^180^
VEGF*	Angiogenic cytokine that promotes hypoxic microenvironment.	Neutralization or inhibition increases efficiency of TB treatment regimens by disrupting the granuloma thus promoting drug penetration and *Mtb* killing in human, rabbit and zebrafish models.	^181,182^
IL-2	Aids proliferation of antigen-specific CD4+ and CD8+ T, activates the JAK-STAT signalling pathway for gene transcription of cell growth and survival genes.	Recombinant IL-2 supplementation with anti-TB drugs improved immunity status and promoted sputum smear conversion to negative, with reduced INF-γ production and low skin response to *Mtb* antigens (response is likely mediated by regulatory T cells).	^183^
IL-4*	Downregulates INF-γ production and mediates cytotoxicity and fibrosis.	Genetically deficient *IL-4*^*−/−*^ mice successfully eradicated *Mtb* infection following reconstruction with recombinant IL-4, and has the potential to be adjunctive to standard TB regimens.	^184,185^
IL-7*	Enhances T-cell memory, upregulates IL-17 production, downregulates TGF-β, aids in DC activation.	*Mtb* mouse models demonstrated increased survival and bacilli clearance when administered in conjunction with BCG vaccine.	^186,187^
IL-10*	Immunoregulatory cytokine with Th2-modulatory effects.	Inhibition of IL-10 in conjunction with anti-TB drugs effectively improved disease outcome and drug efficiency.	^188^
IL-12*	Strong inducer of INF-γ production in antigen-stimulated CD4+ T cells, essential for protective immune response to intracellular pathogens.	Administration to *Mtb*-infected mice decreased viable bacilli load in lymphoid organs. One case study provided evidence of successful response to anti-TB drugs only following treatment with IL-12 for 3 months in a patient that was previously refractory to anti-TB treatment.	^189,190^
IL-15*	Aids proliferation and survival of CD8+ T cells, strengthens immune memory.	BCG-vaccinated IL-15 transgenic mice displayed resistance against *Mtb* infection, thus acting as an immune adjuvant to increase efficiency when administered with BCG vaccination.	^186,191^
IL-23*	Inducer of INF-γ production and proliferation of activated memory T cells.	Vector-mediated intratracheal delivery in mice reduced bacilli load and inflammation.	^192^
IL-24*	Activated CD8+ T cells, increases INF-γ production, activates neutrophils and increases IL-12 production	*Mtb* mouse models demonstrate protective response upon administration of exogenous IL-24.	^193^
IL-37*	Antiinflammatory cytokine, broadly suppresses innate and adaptive immunity.	BCG-infected transgenic IL-37 mice displayed reduced bacilli load and tissue damage in the lung, with reduced frequencies of regulatory T cells and Th17 cells.	^194^
Cytokine modulators			
RhIL-2	Cytokine adjunctive therapy is thought to restore the immune response and modulate the immunologic status in favour of the host by promoting CD4+ and CD8+ T-cell proliferation, and by activating gene transcription pathways of cell growth and cell survival genes.	Study of adjunctive recombinant human interleukin-2 therapy in patients with MDR-TB	^74,183,195,196^
Pascolizumab	The IL-4 cytokine is known as an immunosuppressant molecule which impairs the immune system’s ability to clear *Mtb* infection. Thus, pascolizumab, an anti-IL-4 monoclonal antibody is thought to strengthen the immune response and benefit the host’s ability to clear infection.	Safety and Efficacy of Blocking IL-4 with Pascolizumab in Patients Receiving Standard Therapy for Pulmonary Tuberculosis	^185,197^

*INF-γ* interferon-γ, *TNF-α* tumor necrosis factor-α, *GM-CSF* granulocyte/macrophage-colony stimulating factor, *VEGF* vascular endothelial growth factor, *Ang* angiopoietin, *IL* interleukin, *DCs* dendritic cells, *CD* cluster of differentiation, *JAK-STAT* Janus tyrosine kinase-signal transducer and activator of transcription, *TGF-β* transforming growth factor-β; BCG = Bacille Calmette Guerin, *CFU* colony-forming units, *MGIT* Mycobacteria Growth Indicator Tube

^a^No clinical trial data as evidence for therapeutic intervention potential and outcome in TB disease

Cytokine HDT approaches that have received considerable attention in TB involve IFN-γ or modulation of TNF-α. In particular, aerosol administration of recombinant IFN-γ-1b as supplement to DOTS for patients with cavity PTB was evaluated in a phase-II trial. Results demonstrated favourable immunomodulation by reducing inflammatory cytokines at the site of disease and accelerated *Mtb* sputum clearance (Table 2).^93^ More recent pre-clinical data have however illustrated the propensity for exacerbated lung infection and deleterious effects of increased IFN-γ production by CD4 T cells in murine models.^98^ While TNF-α stimulates monocytes/macrophages and maintains granuloma integrity, high levels may exacerbate pathology. Thus, approaches that decrease TNF-α have been favoured with the rationale of restricting pathology or destabilizing fibrotic granulomas to improve drug penetration^99^ (Table 2). TNF-α-blockers routinely used for treating inflammatory bowel disease and arthritis (such as etanercept) demonstrated some benefit in TB, while TNF-α-antibodies (such as infliximab and adalimumab) have shown success in advanced TB disease.^101–102^ In contrast, a recent meta-analysis indicated that the risk of TB may be significantly increased in patients treated with TNF-α antagonists, and may be evoking more harm than good in majority of patients.^103^ Therefore, despite some promising therapeutic outcomes, the use of IFN-γ and TNF-α modulating agents remains controversial, ultimately due to their interactions being both synergistic and antagonistic.

Considering their involvement in highly complex networks, the therapeutic impact of cytokines is often challenging to predict. This reiterates the importance of critically evaluating dosage systems to ensure optimal benefit to recipients. Additionally, despite promising outcomes of some cytokine-based therapies, employment of such strategies may be restricted by high cost, potential toxicity and role in immunopathology.^104^ Lastly, it has become increasingly evident that single-cytokine HDTs are often inadequate during the initial phase of therapy, and thus requires further exploration for combinatory cytokine therapy options.

### Statins and other drugs

Statins are well-known for their lipid-lowering, immunomodulatory and anti-inflammatory activities. These effects are achieved via inhibition of HMG-CoA (β-Hydroxy β-methylglutaryl-CoA) reductase enzymes, which are essential in lipid metabolism and inflammatory pathways. Since the lipid-rich macrophage is a favourable environment for *Mtb* persistence, statins reducing intracellular lipid accumulation thus limits bacterial growth. Moreover, statins enhance phagosome maturation and autophagy (Table 1).^106–107^ The StAT-TB trial is investigating the safety, tolerability and pharmacokinetics of pravastatin co-administered with standard TB treatment. The ability of pravastatin adjunctive therapy to shorten the time to sputum culture conversion and improve lung function will also be tested in a second phase of this trial (NCT03456102; Table 1). Of particular interest is the drug−drug interactions between statins and anti-TB drugs such as rifampicin and isoniazid.^108^ The associated adverse events such as myopathy and rhabdomyolysis are often associated with non-adherence and unsuccessful treatment, making it particularly important to select statins with no known drug interactions with TB antibiotics.

Another drug, auranofin, is an organogold compound that induces transcription of heme-oxygenase-1. This inducible heme-degrading enzyme exerts anti-inflammatory properties and decreases free radical production, while enhancing oxygen-mediated killing and bactericidal activity in TB disease. A trial in South Africa is currently recruiting TB patients to test the safety and efficacy of auranofin as an adjunctive TB HDT (NCT02968927; Table 1).

Within the scope of cancer management, cell-based therapies are receiving growing interest. Indeed, such strategies may form a good template for HDTs in the TB field. As reviewed by Rao et al., in the context of MDR-TB, adoptive cell therapies and screening techniques could identify useful non-cross-reactive *Mtb* target-specific T-cell receptors (TCRs). These TCRs, in turn, may be transferred into recipient effectors (such as NK or T cells), theoretically giving rise to genetically modified therapeutic cellular products.^109^ Indeed, there has also been promising outcomes in MDR/extensively drug-resistant (XDR)-TB patients receiving mesenchymal stromal cells (MSC) as a single infusion of bone marrow-derived autologous MSC.^110,111^ These MSC are well-known for their safety, anti-inflammatory and immunomodulatory properties and may thus also be applicable to various forms of TB disease.^109,112^ Additionally, microRNAs (miRs) have been implicated as potential adjunctive HDTs to regulate immune responses in TB and improve outcome. This approach holds promise by using miR for repairing and replenishing miR stores or administering anti-miRs to inhibit rogue miR that may otherwise induce pathology.^113,114^ Although having solid theoretical foundation and promising outlooks for the future, cell-based therapies and miRs in the context of TB remains in its infancy, with much still to be uncovered.

Autophagy-activating compounds may represent promising adjunctive therapies against TB disease. A review by Paik et al. discusses autophagy mediators targeting VDR signalling, the AMPK pathway, sirtuin 1 activation and nuclear receptors.^115^ Autophagy-targeting small molecules have shown promise in the context of *Mtb* infection. In pre-clinical testing, gefitinib (targeting EGFR), fluoxetine (a serotonin reuptake inhibitor), baicalin (a herbal medicine targeting the PI3K/Akt/mTOR pathway) induce autophagy and enhance intracellular *Mtb* clearance.^116,117^ Indeed, antimicrobial drugs such as loperamide, verapamil and standard anti-TB drugs (such as INH and pyrazinamide) themselves promote autophagy and may work synergistically with autophagy-inducing small molecules as adjunctive therapy to standard treatment for TB patients.^115,118–120^

Although falling beyond the classification of HDT, antibiotics (such as doxycycline; NCT02774993; Table 1^121,122^) and vaccine strategies may modulate immunity, and have been proposed as potential adjunctives to the TB treatment regimen. There is evidence supporting beneficial effects of Bacille Calmette-Guérin (BCG) re-vaccination in adolescents and adults. Results indicate that BCG re-vaccination reduces the rate of sustained QuantiFERON (QFT) conversion and displays improved long-term innate or trained immunity and adaptive responses, thus leading to effective control of mycobacterial infection.^123–129^ A comprehensive review by Schaible et al. in 2017 evaluates strategies to improve vaccine efficacy against TB by targeting innate immunity.^130^ Here, they propose that short-term modulation of the local immune response to BCG vaccination may result in long-term protective immunity against *Mtb* infection. Examples of interventions for such modulation may involve regulating neutrophil, Treg and MDSC recruitment to the vaccination site, preventing disadvantageous cell death pathways, modulating vaccination-induced inflammatory responses, and regulating anti-inflammatory cytokine profiles (e.g. IL-10).^130^ These strategies are aimed at processes that would otherwise negatively influence T-cell priming, function and proliferation upon vaccination; and many of which are in fact analogous to the rationale of HDT strategies discussed here.

## Conclusion

While the outcome of some trials has been met with anticlimactic conclusions, emerging evidence outlined in this review suggests that the TB field is making steady progress in identifying beneficial HDTs across a broad range of drug classification and mechanistic activity. Building on these data, it is hoped that future investigations will translate into meaningful, effective clinical developments.

According to the therapies discussed in this review, we propose that certain HDTs will be of particular relevance to a specific TB infection/disease group. In this regard, we recommend the following HDT strategies to be most appropriate against active TB and associated forms of TB (such as, TB-IRIS, TB-induced pulmonary diseases and extrapulmonary TB): eicosanoid modulators (NSAID and lipoxygenase inhibitors), inflammatory mediators (corticosteroids and tyrosine kinase inhibitors), metformin, ICIs, cytokine modulating therapy, statins, auranofin, cell-based therapies, miR and autophagy modulating drugs.

As TB preventative therapy, HDTs could, for example, alter bacillary cell entry or enhance anti-mycobacterial properties of lung phagocytes. Ideally, this would prevent infection, while also averting disease development in latently infected individuals. For TB contacts, those in high-exposure settings, recent *Mtb*-infected individuals and LTBI, we propose promising outcomes associated with host-strengthening preventative strategies, including vitamin supplementation, PBA (acting in synergy with vitD), NAC and BCG re-vaccination. We further propose HDTs to supplement new vaccines which may include: PDE-i to regulate effects of immunosuppressive subsets such as MDSC, ICIs to modulate cell death pathways, cytokine therapy to regulate anti-inflammatory cytokine profiles and perhaps eicosanoid and inflammatory mediators to modulate vaccine-induced inflammatory responses and potentiate vaccine-specific responsiveness and durability.

One of the major shortcomings of HDTs includes off-target and associated side effects. These drawbacks require further evaluation against the backdrop of other aspects such as storage stability, delivery method, formulation and timing of administration at different phases of *Mtb* infection and TB disease. In this context, several HDTs remain highly controversial and require more investigation into their potentially severe off-target and associated effects. Two major examples include, ICIs and cytokine therapies. Although offering preventative HDTs to latently infected individuals remains a promising avenue, distinguishing latent infection from early active TB is however, challenging in high-exposure regions. Applicability of HDTs to MDR-TB, TB treatment shortening, TB/HIV and TB-derived lung diseases, although highlighted in some studies, have not been considered for all HDTs. This leaves much to be answered in the context of the TB spectrum. Furthermore, many theoretically sound approaches remain in their infancy in the TB field and require further investigation, hopefully showing promise and advancing to clinical trial status. Here we propose keeping a watchful eye on autophagy modulators, cell-based and miR therapies.

Considering the spectrum of TB disease formats and complexity of host immunity, adjunct HDT is unlikely to be efficient as a ‘one-size-fits-all’ approach. Even so, personalized medicine is also not feasible in high-burdened TB regions, making a case for a precision medicine approach, tailored to phenotypic disease groups. Therefore, developing biosignatures translating into an efficient, rapid, point-of-care pre-screening test is of great interest. In this way, immune profiling or patient stratification according to the degree of lung involvement or risk of disease relapse, will be a game changer for TB treatment strategies. The field remains inspired in the face of our ambitious goal to eradicate TB disease, and the even greater aspiration of preventing *Mtb* infection. These goals are hoped to be achieved through clever strategies involving multimodular approaches, including implementation of adjunct HDT as standard of care for TB patients.
